# Flat Terahertz Reflective Focusing Metasurface with Scanning Ability

**DOI:** 10.1038/s41598-017-03752-3

**Published:** 2017-06-14

**Authors:** Huan Yi, Shi-Wei Qu, Bao-Jie Chen, Xue Bai, Kung Bo Ng, Chi Hou Chan

**Affiliations:** 1 School of Electronic Engineering, University of Electronic Science and Technology of China (UESTC), 2006 Xiyuan Avenue, Western High-Tech District, Chengdu, 611731 China; 2 State Key Laboratory of Millimeter Waves, Partner Laboratory in City University of Hong Kong, Kowloon, Hong Kong China

## Abstract

The ability to manipulate the propagation properties of electromagnetic waves, e.g., divergence, focusing, holography or deflection, is very significant in terahertz applications. Metasurfaces with flat structures are attractive for achieving such manipulations in terahertz band, as they feature low profile, lightweight, and ease of design and installation. Several types of terahertz reflective or transmitting metasurfaces with focusing function have been implemented recently, but none of them can provide scanning ability with controllable focus. Here, a flat reflective metasurface featuring controllable focal shift is proposed and experimentally demonstrated. Furthermore, the principle of designing a focus scanning reflective metasurface is presented and the focusing characteristics are discussed, including focus scanning along a line parallel or orthogonal to the metasurface with a large bandwidth. These interesting properties indicate that this flat reflective metasurface could play a key role in many terahertz imaging and detection systems.

## Introduction

Metasurfaces have attracted substantial interest in recent years because they have excellent ability to manipulate the propagation properties of electromagnetic waves. Multi-functional devices have been developed based on metasurfaces, such as beam splitters^1, 2^, polarizer^3^, reflectarrays^4^, absorbers^5^, filters^6^, hologram^7^, lenses^8, 9^, and mirrors^10–12^. Metasurfaces with focusing ability are one of the key devices in many applications^8–12^, and it is well known that transmitting and reflective metasurfaces (REMs) are the most common focusing devices. In general, transmitting metasurfaces or planar lenses with subwavelength phase-gradient elements are usually composed of multilayered structures^8, 13^, which are difficult to be fabricated and suffer from alignment errors between different layers. The ultrathin lenses composed of a single layer of plasmonic antennas are also reported, but they have very low efficiency^14–17^. In comparison, the REMs are more compact with smaller thickness, higher efficiency^10, 11, 18^, and ease of fabrication owing to their single-layer patterns with a grounded substrate.

As we know, focus scanning is urgently in demand for terahertz (THz) applications like imaging, sensing, and communication^19–21^. However, among all the methods for focus scanning, the time-consuming and complicated mechanical ones^22–24^ are still dominant in the THz region. In the microwave region, the fast scanning of antenna arrays can be realized using the phased array technique^25, 26^. This concept has been further extended to the dynamical control of the THz waves, but it is bulky and operates only in one-dimensional (1D) focusing case, acting like a cylindrical lens as presented in ref. 27. As an alternative, devices with frequency-controlled focus scanning are good candidates for these applications because they feature lower cost and less complicated structures. Such devices have been reported in the literature, e.g., a plasmonic waveguide^28^ or rectilinear leaky-wave lenses^29^. However, they are either only in 1D case or difficult to be excited, especially in the THz region. Furthermore, the focal positions of these designs cannot be controlled in the whole operating frequency band.

In this paper, a frequency-controlled THz flat focusing REM with subwavelength phase-gradient elements is proposed. Instead of achieving focus scanning through the uncontrollable dispersion of a waveguide or element^14, 28^, the proposed flat REM is carefully designed by artificially controlling the dispersions of the elements and desired phases on its surface. Hence, the focal position is well controlled by varying the operating frequency. To demonstrate the focus-scanning ability, the design, simulation, fabrication and characterization results of the flat REM are presented in this paper. The REM element is composed of a ring and an I-shaped resonator placed on top of a grounded dielectric substrate. The REM is realized by using standard micro-fabrication method. Consequently, the focal points of the proposed REM are moved along the direction parallel to the REM with respect to frequency. Furthermore, the design principle can be extended to implement longitudinal scanning or a wideband REM without focus dispersion. The focusing REMs presented here have great prospect in the applications of fast THz imaging, 3D imaging, and detection.

## Materials and Methods

### Element configuration

**Figure 1:**
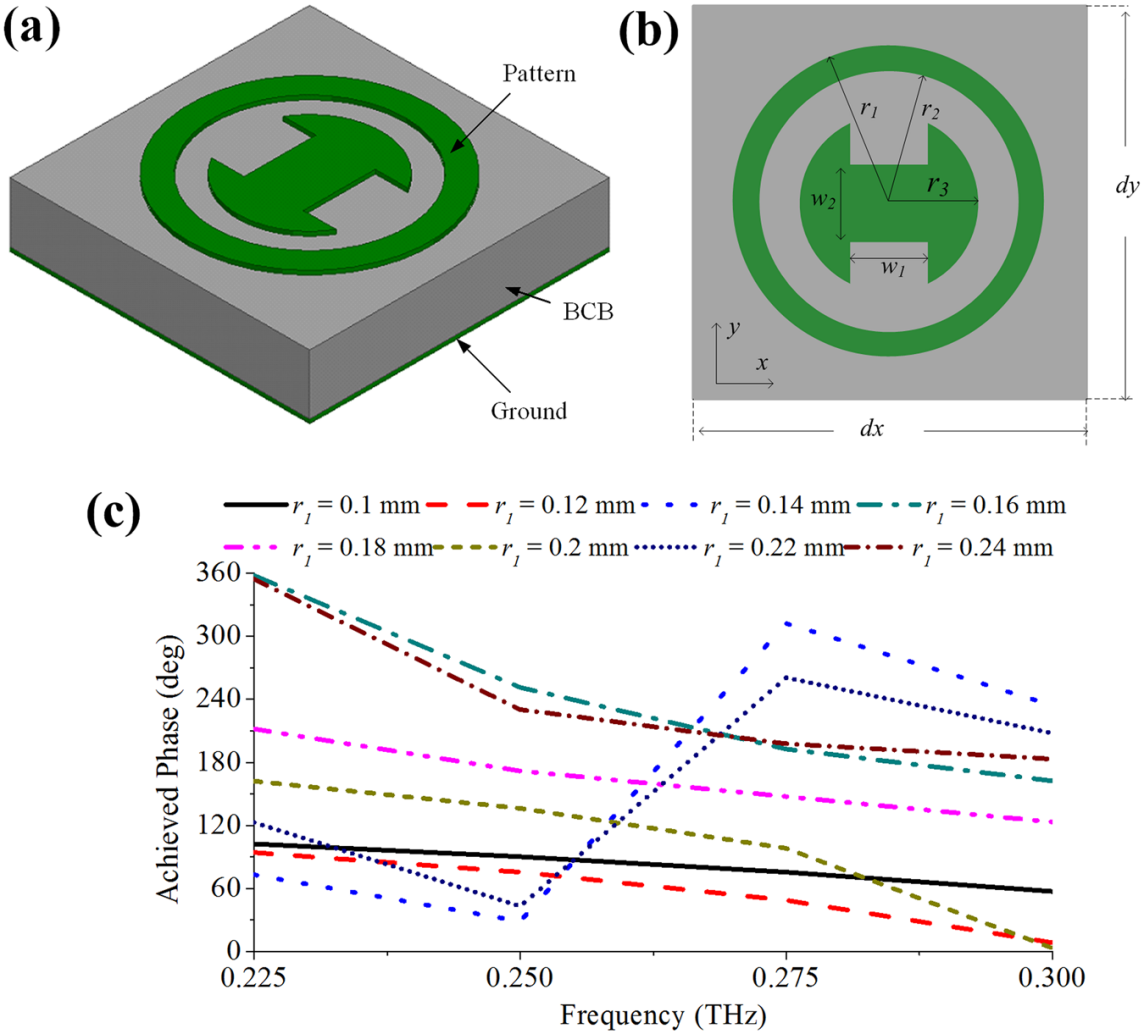
Element geometry. (**a**) 3D view. It consists of three layers, i.e., a ground layer, a 100-µm-thick BCB layer, and the pattern layer with 1-µm-thick aluminum on the top of the BCB layer. (**b**) Top view, the pattern layer is composed of a ring and an “I” shaped resonator. (**c**) Achieved phase versus frequency by the element. *d*_*x*_ = *d*_*y*_ = 0.5 mm, *r*_*2*_ = *r*_*1*_ − *s, r*_*3*_ = *r*_*2*_ − *s*, *w*_*1*_ = *nr*_*3*_, *w*_*2*_ = 2·(1-*n*/1.7)·*r*_*3*_, *s* = 30 µm, *n* = 1.

The proposed REMs are comprised of a group of elements with different sizes. The configuration of the employed element is shown in Fig. 1a,b, which consists of a ground layer, a 100-µm-thick benzocyclobutene (BCB) layer, and a pattern layer with 1-µm-thick aluminum on the top of the BCB layer. Figure 1c shows the reflection phases of the REM elements versus frequency with x-polarized incidence. It can be seen that the slopes of the phase curves change slightly for elements with different *r*_*1*_, and the largest phase difference between 0.225 and 0.3 THz is about 300°.

### THz REM design

To successfully implement the proposed concept of the THz REM, a database of the phase response of the element with different physical parameters is built as the first step. Since not all parameters are critical to tune the reflection phase, only three, namely, *r*_*1*_, *s* and *n* are chosen to map the physical sizes of the unit cell to the achieved reflection phase, as given in Section II of the Supplementary Information. In the multi-dimensional database, the index of each element indicates the physical size of the unit cell and its corresponding reflection phase value. Then, the THz REM can be designed based on the database after the source direction is provided. To design the THz REM, the unit cells with proper physical dimensions are carefully selected from the well-prepared database by multiple frequency phase matching method, i.e., simultaneously matching the achievable reflection phases to the desired values of each element at 0.225, 0.250, 0.275, and 0.300 THz. In this process strict rules are set on the element performances which, however, can simplify the metasurface design in return.

### Fabrication

The THz REM was fabricated by standard micro-fabrication method in the State Key Laboratory of Millimeter Waves, Partner Laboratory in City University of Hong Kong (PSKLMW). First, polymer BCB from Dow Chemical Company, was spin-coated and cured (at 270 °C for 2 hours) onto a flat aluminum plate. Aluminum was chosen for better adhesiveness to the BCB layer, and the thickness of the BCB layer was set at 100 µm which was determined by the spinning speed and time (1200 rpm, 2 min, spin coating for 3 times). Then, a 1-µm-thick aluminum film was deposited onto the BCB layer by thermal evaporation. Finally, the aluminum pattern was fabricated by photolithography process followed by aluminum wet etching. Photograph of the zoom-in view of a section of the fabricated THz REM prototype is shown in the inset of Fig. S3 in the Supplementary Information.

### Measurement setup

To obtain the experimental results, a measurement setup is built in PSKLMW (seen in Section III of the Supplementary Information). The system can cover a frequency range from 0.220 to 0.330 THz, and the platform with a receiving probe can be moved along the x and z axes which are parallel to the working bench to map the two-dimensional (2D) reflected power distribution. Meanwhile, the horn antenna is located at 760 mm far from the REM, i.e., in its far-field region, and then the incident waves can be regard as the plane waves.

## Results and Discussion

### Principle of THz focus scanning REM

In order to focus the incident plane waves, the flat REM surface must undergo a spatially varying phase shift. The following expression governs the relationship between the desired phase Φ*(x*_*n*_ *)* and the focal position, as well as the direction of the incident waves *θ*:

1 $${\rm{\Phi }}({x}_{n})={k}_{0}[\sqrt{{F}^{2}+{({x}_{n}-{x}_{0})}^{2}}-F-{x}_{n}\,\sin (\theta )]+{{\rm{\Phi }}}_{0}(f)$$

where *F* is the focal length, *x*_*0*_ is the focal position along the x axis, *x*_*n*_ is the position of the nth element, and *k*_*0*_ is the free-space wave number. In general, the desired phase Φ(*x*_*n*_) is fulfilled by choosing appropriate elements only at the center frequency. Phase errors are certainly introduced at other frequencies due to uncontrollable dispersion of the elements. In this article, to obtain a controllable focus scanning REM, the desired phase compensation can be regarded as a function of focal position *x*_0_, and the operating frequency *f*, i.e., Φ(*x*_*n*_, *f*, *x*_*0*_).

**Figure 2:**
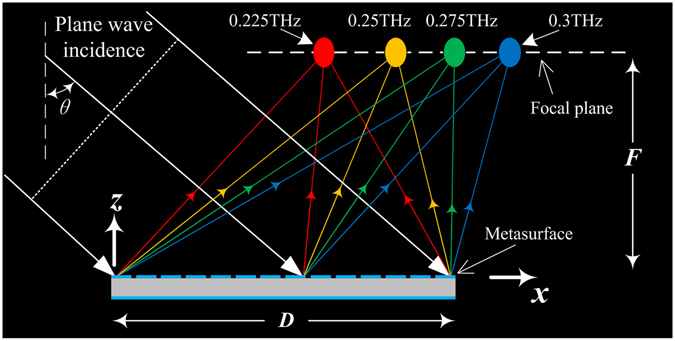
Illustration of the operating principle of the focus scanning REM. The plane waves incident on the metasurface and the reflected waves at different frequencies are focused at different positions.

The configuration of the focus scanning REM is presented in Fig. 2. It is illuminated by the x-polarized incidence with *θ* = 45°, and then the reflected waves are converged at the focal points. The focal positions in the focal plane with respect to the frequencies are presented in Fig. 2. To clearly illustrate the desired phase against *f* and *x*_*0*_, four focal positions at four different frequencies are chosen: *x*_*01*_ = 13.5 mm at *f*_*1*_ = 0.225 THz, *x*_*02*_ = *x*_*01*_ + *Δx* at *f*_*2*_ = 0.25 THz, *x*_*03*_ = *x*_*01*_ + 2*Δx* at *f*_*3*_ = 0.275 THz, *x*_*04*_ = *x*_*01*_ + 3*Δx* at *f*_*4*_ = 0.3 THz, and *F* = 40 mm, where *Δx* is the lateral-shift spacing between the focal points of adjacent selected frequencies. Here, the sign of *Δx* indicates the direction towards which the focus is moved (*Δx < *0 for −x direction and *Δx* > 0 for +x direction) and *Δx* = 0 means that the focus is fixed with the variation of frequency. According to these data, the desired phases Φ(*f*_*1*_ *,x*_*01*_), Φ(*f*_*2*_ *,x*_*02*_), Φ(*f*_*3*_ *,x*_*03*_) and Φ(*f*_*4*_ *,x*_*04*_) can be obtained at each position on the REM aperture according to Equation (1).

**Figure 3:**
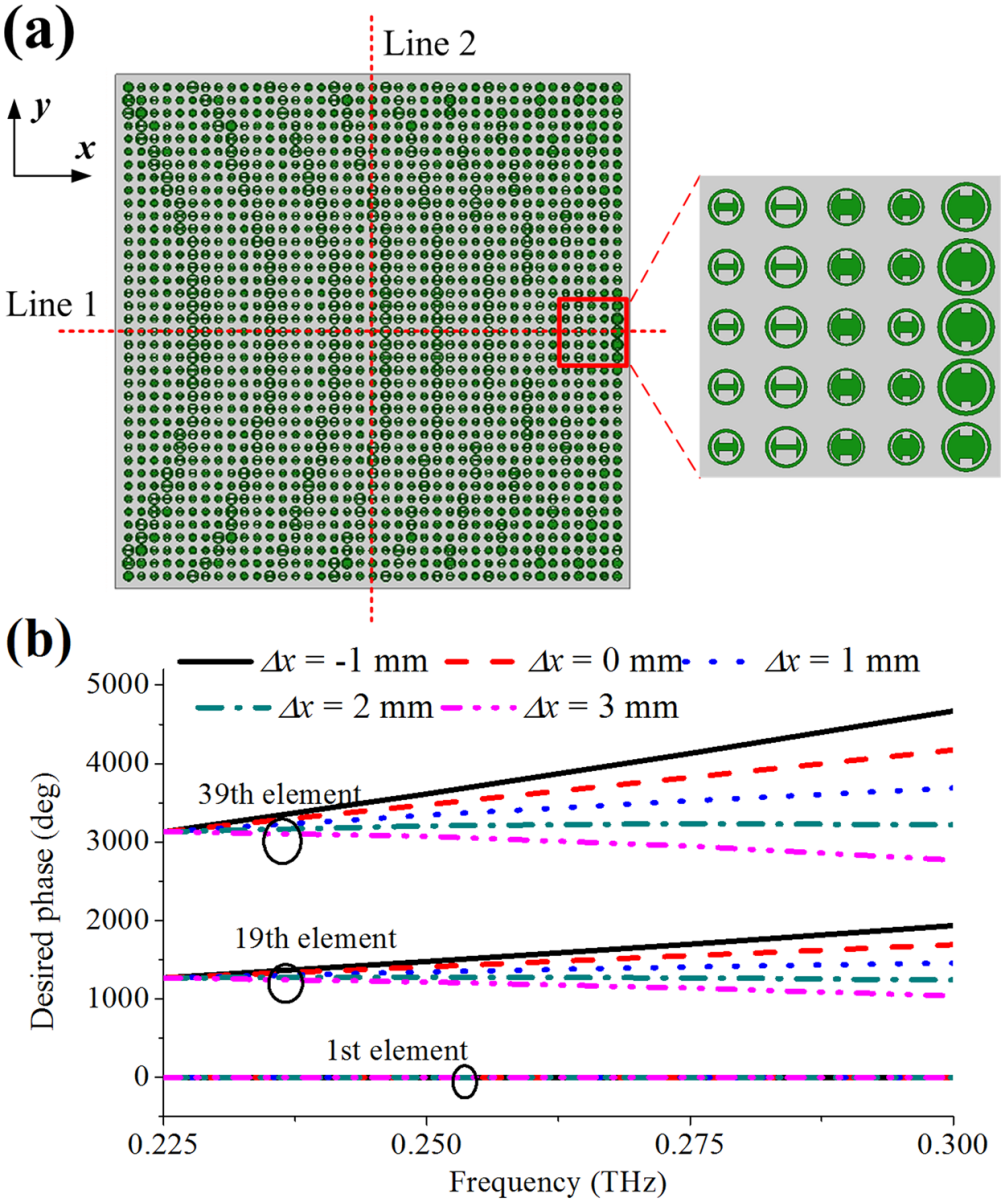
(**a)** Element arrangement of the THz REM. The origin of the coordinate system is located at the center of the first column of unit cells, and the z axis is normal to the xy plane. Two referenced lines, i.e., Lines 1 and 2, are the orthogonal central lines on the surface and the focus points are located above but parallel to the xz plane and scan along the x axis. (**b**) Desired phases for different positions on the REM aperture with different focus-shift spacing. Note that the desired phase can be transformed into 0~2π region by +/− 2mπ, where m is an integer.

The physical dimensions of the REM are designed to be 19.5 × 19.5 mm^2^, consisting of 39 × 39 elements along the x and y directions, respectively. Figure 3a shows the element arrangement on the THz REM. The desired phases of the 1st, 19th and 39th elements along Line 1 with different values of *Δx* are presented in Fig. 3b. Note that the phase curves of the 1st element are overlaid as a reference. It can be seen that the phase curves with different slopes are required at different positions on the REM aperture. Meanwhile, when *Δx* increases from −1 to 3 mm, the modulus of the slopes of these curves gradually decreases to zero and then increases again. The desired phase curve is flattest as *Δx* = 2 mm, which indicates that the elements with approximately parallel phase responses can meet the phase requirement.

### Phase Compensation by Elements

**Figure 4:**
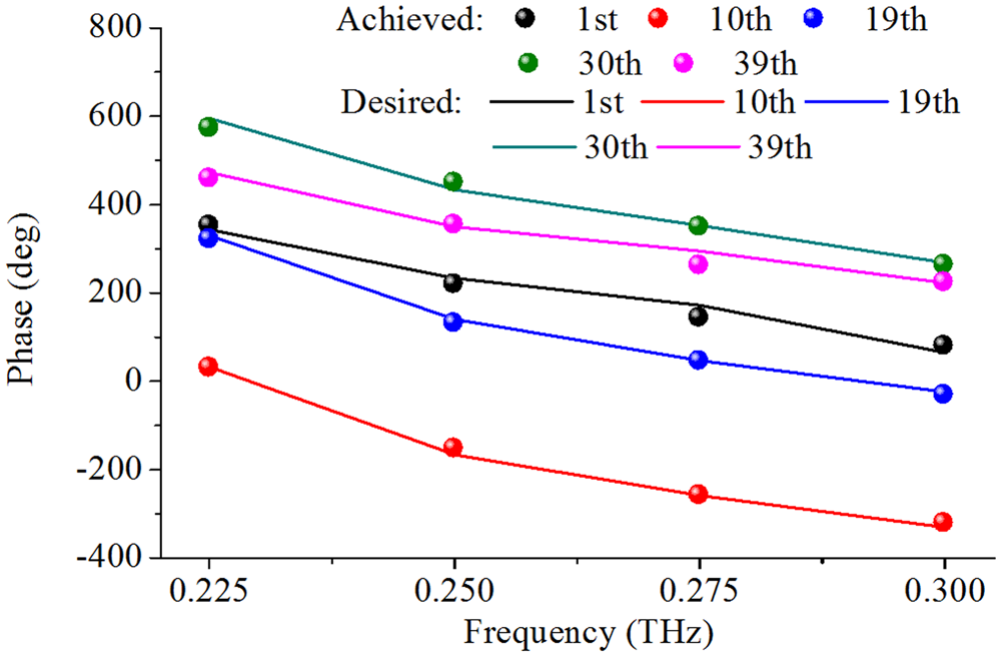
Achieved phase matching by the elements at five different positions along line 1 on the REM aperture. The solid lines are the desired phases Φ(*x*_*n*_, *f*, *x*_*0*_) which are obtained according to Equation (1), and the markers denote the reflection phases achieved by the selected elements for phase compensation from the database.

In the design of focus scanning REM, *Δx* = 2 mm is chosen as the lateral-shift spacing, and in this case the variation of phase-curve slope is smallest for different positions as presented in Fig. 3b. Therefore, the focal positions of the THz REM at 0.225, 0.25, 0.275, and 0.3 THz are designed at (*F, x*_*0*_), where *F* = 40 mm and *x*_*0*_ is equal to 13.5, 15.5, 17.5, and 19.5 mm, respectively. After these parameters are determined, the desired phase Φ(*x*_*n*_, *f*, *x*_*0*_) can be calculated by Equation (1), then appropriate elements are chosen from the database to fulfill the desired phase compensations at each position on the REM aperture at all frequencies simultaneously. The overall configuration of the focus REM is shown in Fig. 3a. The phase compensations achieved with different elements are shown in Fig. 4, it can be seen that good matching between the desired and achieved phases is obtained at all frequencies and the phase difference between them is 11° for each element on average. Note that other value of lateral-shift spacing *Δx* is achievable but maybe with larger phase errors or larger phase range of the elements is demanded.

### Experimental characterizations of the focus-shift REM

**Figure 5:**
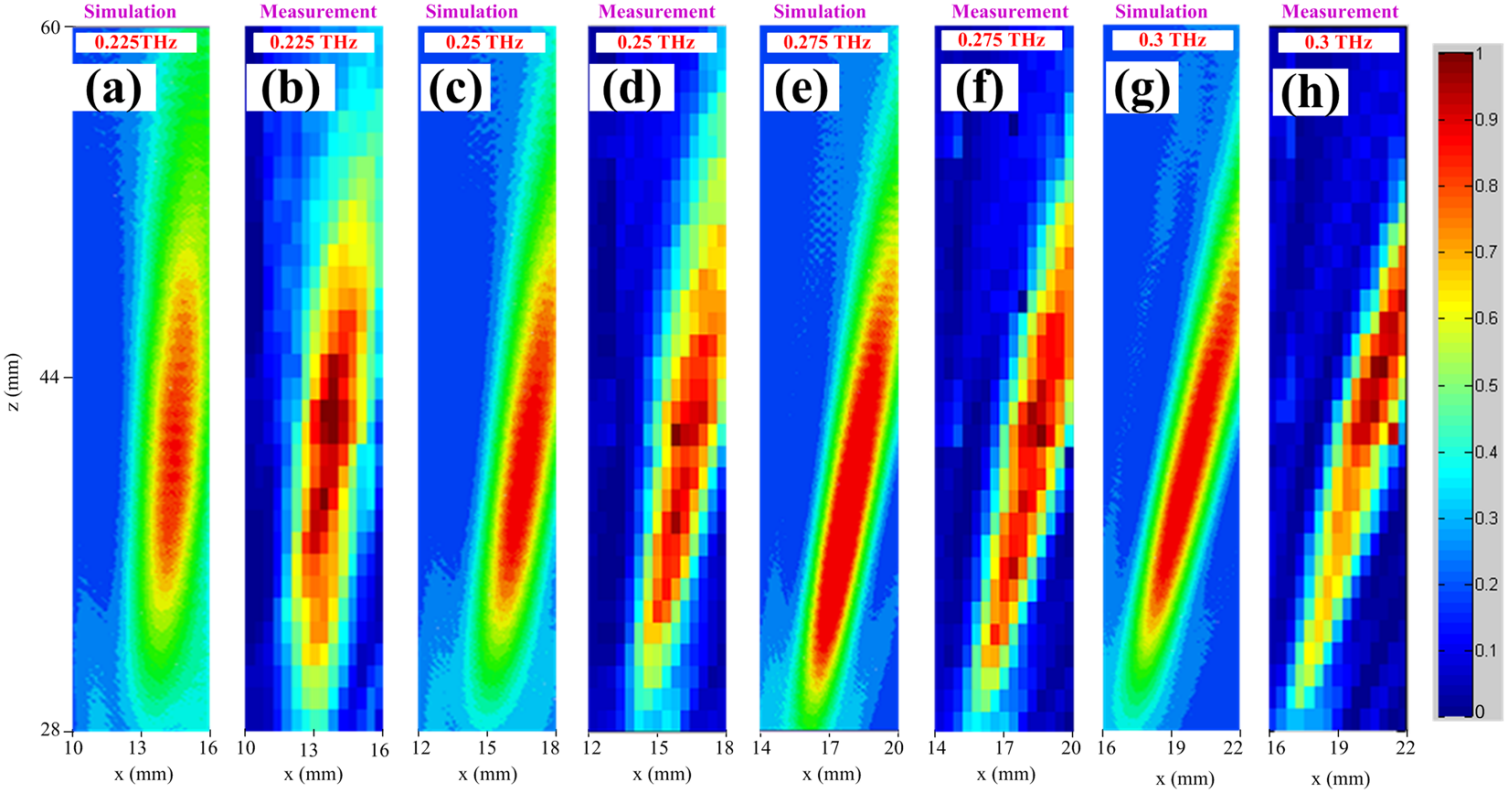
Simulated and measured normalized power intensity distributions in the xz plane. The region along the x and z axes are 6 and 32 mm, respectively. (**a**) Simulated and (**b**) measured results at 0.225 THz with *x* ranging from 10 to 16 mm and *z* from 28 to 60 mm. (**c**) Simulated and (**d**) measured results at 0.25 THz with *x* ranging from 12 to 18 mm and *z* from 28 to 60 mm. (**e)** Simulated and (**f**) measured results at 0.275 THz with *x* ranging from 14 to 20 mm and *z* from 28 to 60 mm. (**g**) Simulated and (**h**) measured results at 0.300 THz with *x* ranging from 16 to 22 mm and *z* from 28 to 60 mm. The measurement results with steps along the x and z axes are 0.4 and 1 mm, respectively.

**Figure 6:**
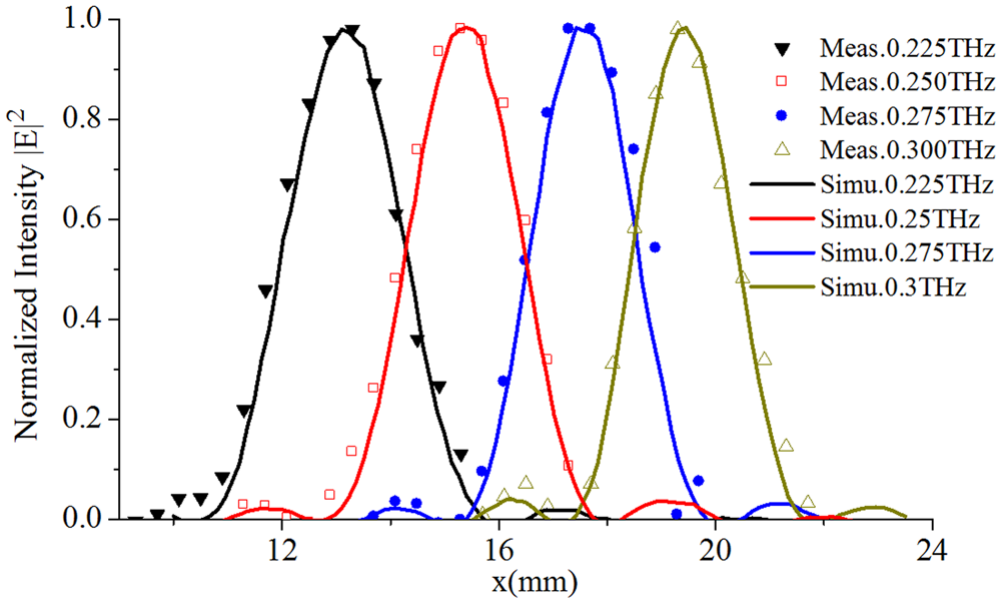
Simulated and measured normalized power intensity distribution along the line with *y* = 0 mm, *z* = 40 mm. In the figure, the power distribution within a range from 9 to 24 mm is shown. All measured and simulated results are normalized to their maximum values. The measured results at different frequencies are shown by different types of markers, and the simulated ones are by the smooth curves. The simulated results at four frequencies are given, i.e., 0.225, 0.25, 0.275, and 0.3 THz, while the measurements are performed with a step of 0.4 mm by a pair of the VDI frequency extenders from 0.22 to 0.33 THz (Virginia Diode Inc.), respectively.

Full-wave numerical validation is performed using High-Frequency Structure Simulator (HFSS). The proposed flat REM is shown in Fig. 3a, which has a focal length of 40 mm, and a square aperture with sizes of 19.5 × 19.5 mm^2^. For the plane waves incident at an angle of 45°, the reflection waves are focused at different positions for different operating frequencies. The measured results agree well with that of simulations as shown in Figs 5 and 6. Figure 5 shows the normalized power intensity distribution in the xz plane with different x values at different frequencies. The measured and simulated results show that the focal points at different frequencies are located in different positions as desired. A 1D plot of the power intensity distribution along the x axis at *z* = 40 mm and *y* = 0 mm is shown in Fig. 6, and it can be seen that the measured results agree well with simulation which clearly demonstrate the focal-scanning ability of the flat REM.

### Focus scanning along the z axis

**Figure 7:**
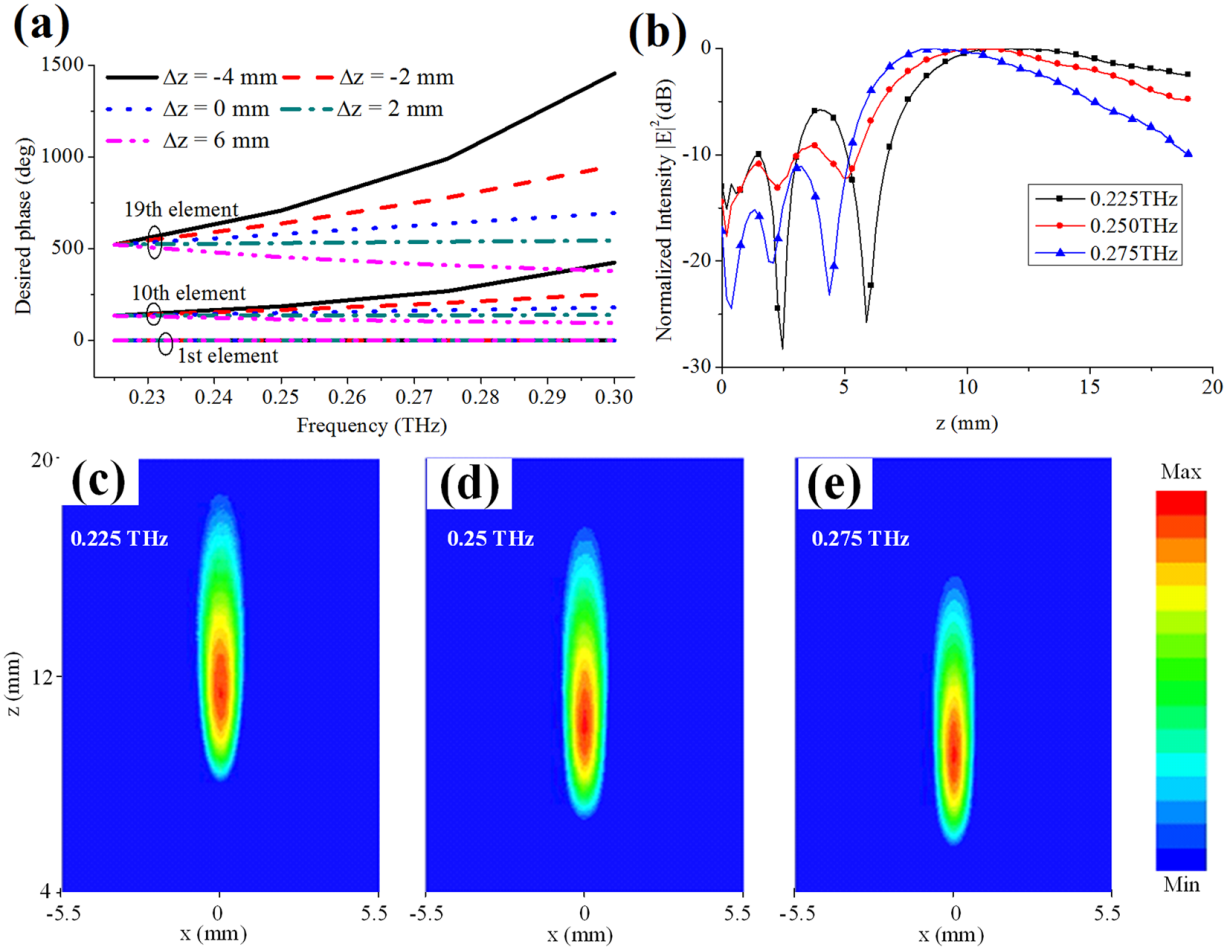
(**a**) Desired phases at different positions on the REM aperture with different focal length shift spacing. The REM is illuminated by the normal incidences, and the focus is located above the REM center with *F*_*o*_ = 14 mm at 0.225 THz. *Δz* is the focal distance between two adjacent frequencies, and the signs “**−**” and “+” represent the focus is moved toward or away from the REM as frequency increases, respectively. The presented three groups of curves are the desired phases at different positions on the REM aperture. Note that this REM has similar topological structure with the previous one as shown in Fig. 3a, and due to symmetry of the REM, here the element number is counted from the REM center along x axis. (**b**) Simulated normalized power intensity distributions of the REM with focuses moved along the z axis against frequencies. The intensity distribution within a range from 0 to 19 mm is shown. All simulated results are normalized to their maximum values. Finally, the simulated results at three frequencies are given, i.e., 0.225, 0.250, and 0.275 THz. Simulated intensity distributions in the xz plane are given at (**c**) 0.225 THz, (**d**) 0.250 THz, and (**e**) 0.275 THz.

Based on the same idea, the desired phase compensation of the nth element can be regarded as a function of focal length *F*, and the operating frequency *f*, i.e., Φ(*x*_*n*_, *f*, *F*), which reveals that the focus is moved along the longitudinal direction as frequency varies. In fact, the metasurfaces are always dispersive, which means that their focal lengths always vary as frequency changes^8, 11, 13, 14, 17, 24, 30^. Meanwhile, it should be noted that the focal points are always moved away from the metasurface with the increase of frequency^8, 11, 13, 14, 17, 24, 30^, which is referred here as a normal-dispersion metasurface. This phenomenon can be understood by the relationship between the desired phase and focus-shift range along the z axis as shown in Fig. 7a. It can be seen that the phase curve is flatter as the focal point is moved away from the metasurface with the increase of frequency in a certain range, while the phase curve becomes steeper with the focal point moving toward the metasurface as frequency increases. In most cases, the phase curves of different elements have small slope variations, as presented in Fig. 1c. Therefore, the focal point always moves away from the metasurface as frequency rises^7, 11, 13, 14, 17, 24, 30^.

Based on our concept, if the elements can provide the desired phase at all frequencies, a REM with an inverse relationship between the focal length and frequency can be achieved which is called anomalous-dispersion REM. For verification, three frequencies are taken into consideration, i.e., 0.225, 0.250, and 0.275 THz. The corresponding focuses are located at *z* = 14, 12, and 10 mm, respectively, with *x* = *y* = 0 mm. The desired phase Φ(*x*_*n*_, *f*, *F*) can be calculated using Equation (1), and the aforementioned element database is utilized to find the elements that can provide the desired phase compensation. The REM consists of 19 × 19 elements with dimensions of 9.5 × 9.5 mm^2^. Full-wave simulations are performed to verify the longitudinal focus scanning ability of the REM, and the results are shown in Fig. 7b. It can be seen that the focal length decreases as frequency increases. The two-dimensional (2D) power intensity distribution in the xz plane is shown in Fig. 7c–e.

**Figure 8:**
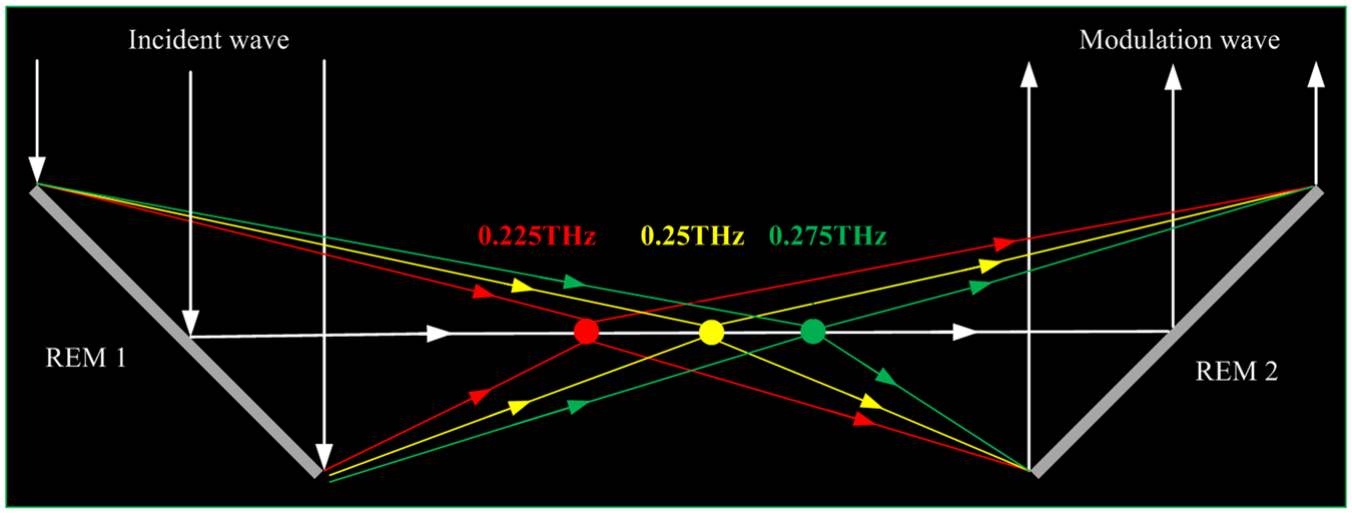
Schematic of the imaging system with two REMs, which have opposite dispersion characteristics. The focal length of REM 1 increases while that of REM 2 decreases as frequency goes up. Therefore, the images of different frequencies represent different layers in the sample, and then a 3D imaging can be obtained.

These characteristics of the two aforementioned REMs have many interesting applications, for instance, by combining a normal-dispersion and an anomalous-dispersion REMs, as presented in Fig. 8, an imaging system can be set up for potential 3D THz imaging. In addition, for larger scanning space coverage, larger phase-curve slope variations are needed; therefore, multi-resonator structures should be included in the element design. Phase responses of the elements with different number of resonators are presented in Section IV of the Supplementary Information. Furthermore, to achieve a wideband REM with a fixed focal position, the focal length *F* and the focal position *x*_*0*_ can be set to constant values with respect to different frequencies. Then, the desired phases Φ(*x*_*n*_, *f*) can be obtained by Equation (1), and suitable elements can be selected from the database to fulfill the desired phases at all frequencies simultaneously. More details can be found in Section V of the Supplementary Information.

## Conclusions

In conclusion, we have proposed and experimentally demonstrated a THz REM with focus scanning ability along a defined line. The simulations and measurements are found to be in good agreement. Theoretically, any lateral-shift spacing can be achieved by meeting the desired phase compensation. Unfortunately, the phase variation ranges of the elements are always limited. Therefore, phase matching between the desired and achievable phases should be optimized by choosing appropriate focus-shift spacing and elements. Moreover, the metasurface characteristics of normal and anomalous dispersion in the longitudinal direction are pointed out, and an anomalous-dispersion metasurface is realized. These designs provide many more degrees of freedom for the THz focusing REM, which is demonstrated to be capable of scanning not only in the parallel direction but also in the orthogonal direction to the REM surface. In fact, scanning in both directions can be simultaneously realized by properly controlling the dispersion and phase compensation of each individual element. The focusing REMs presented here have great prospective applications such as fast THz imaging, 3D imaging, and detection.

## Electronic supplementary material

Supplementary Information
