# Supplementary material for: Flat Terahertz Reflective Focusing Metasurface with Scanning Ability
Source: Sci Rep. 2017 Jun 14;7:3478. doi: 10.1038/s41598-017-03752-3 (PMC5471233; doi:10.1038/s41598-017-03752-3)
Supplement: Supplementary file 1 — Supplementary Information [file 41598_2017_3752_MOESM1_ESM.pdf]

Supplementary Information for

Flat Terahertz Reflective Focusing Metasurface with Scanning Ability

Huan Yi[1,2], Shi-Wei Qu[1*], Bao-Jie Chen[2], Xue Bai[1], Kung Bo Ng[2], Chi Hou Chan[2]

[1]School of Electronic Engineering, University of Electronic Science and Technology of China (UESTC), 2006 Xiyuan Avenue, Western High-Tech District, Chengdu 611731, China.

[2]State Key Laboratory of Millimeter Waves, Partner Laboratory in City University of Hong Kong, Kowloon, Hong Kong, China.

The supplementary information includes:

I. Operating principle of the element

II. Database of the element

III. Measurement setup

IV. Phase responses of elements with different number of resonators

V. Reflective metasurface with fixed focal position in a certain frequency band

VI. Conversion of the desired phase slope

I. Operating principle of the element

The extra phase of the element can be attributed to the dipolar resonance of the structure, which is the same as the conventional metasurface element in microwave and THz regions. Meanwhile, the phase variation can be attributed to the different electric field distributions at different frequencies or with different structures, e.g., Figs. S1 (a) and (b) show the electric field distributions of the element at 0.225 and 0.3THz with r_1=0.19mm. It can be seen that the dipolar resonances are introduced and different electric field distributions are observed at different frequencies, i.e., the fields at 0.225THz are distributed on both the outer loop and I-shaped resonator, and the fields at 0.3THz are dominated by the inner I-shaped dipole. These indicate that the same structures have different phase responses at different frequencies. Meanwhile, Fig. S1 (c) shows the electric field distributions with r_1=0.13mm at 0.225THz, comparing with the distribution in Fig. R1 (a), the electric field on the I-shaped resonator is much weaker, which indicates that different structures have different phase responses at the same frequency.

The phase responses of the element at different incident angles are presented in Fig. S2, it can be seen that the reflection phase variation is not significant with an incident angle of 45°, but when it is increased to 60°, the phase difference is quite large especially at higher frequencies.

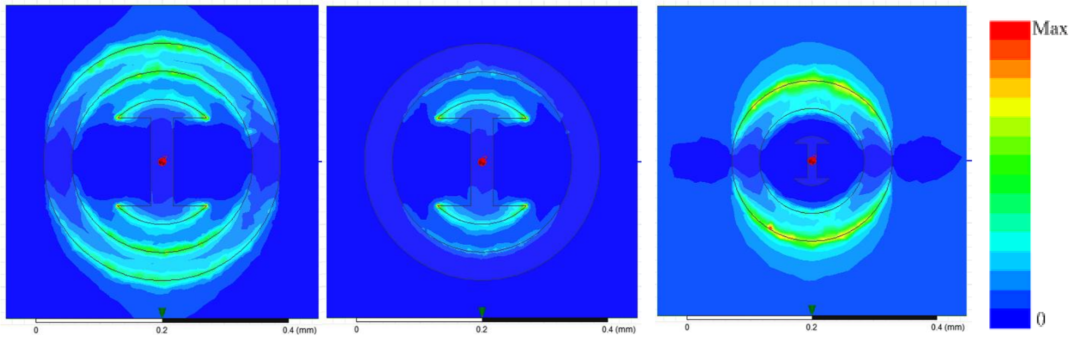

(a) (b) (c)

Fig. S1 Electric field distributions on the surface. (a) r_1=0.22mm at 0.225THz, and (b) r_1=0.22mm at 0.3THz, and (c) r_1=0.13mm at 0.3THz

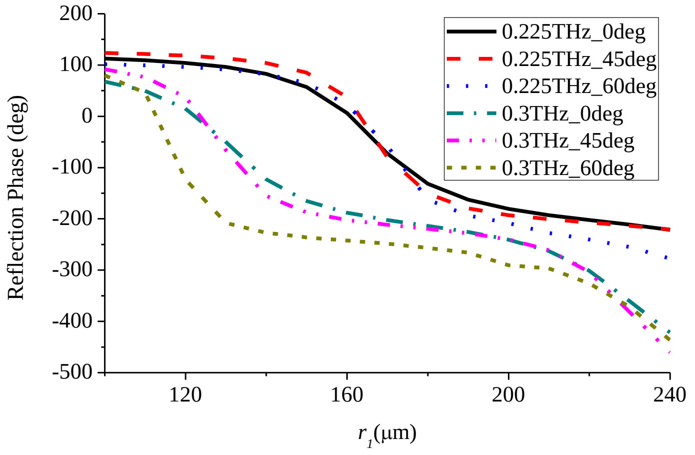

Fig. S2 Phase responses of the element with different incident angles versus r_1

II. Database of the element

A database of the reflection phase of the element is firstly built to map the physical sizes to the reflection phases at 0.225, 0.250, 0.275 and 0.300 THz, respectively. The relationships of the physical parameters are as follows: $r_2 = r_1 - s$, $r_3 = r_2 - s$, $w_1 = nr_3$, $w_2 = 2\cdot(1-n/1.7)\cdot r_3$. Three parameters and the discretized steps are shown in Table I. The parameter r_1 is discretized by a step of 10μm to reduce the required computational time in the full-wave simulations. After a careful parametric sweeping, an interpolation process is performed to obtain more detailed phase values with a step size of 2μm for the first parameter. Then, a three-dimensional database has been established, in which the index of each element indicates the physical sizes of the unit cell and its corresponding reflection phase value, as mentioned in the main content.

Table I. Three swept parameters to build the database of reflection phase at 0.225, 0.250, 0.275 and 0.300 THz.

Parameters	r_1 (μm)	n	s (μm)
Ranges	100~240	0.5~1.4	15~45
Step	10	0.1	5

III. Measurement setup

For THz measurement, a measurement setup is built in PSKLMW as shown in Fig. S3, the signal generator provides the low-frequency input for the multiplier (WR3.4 AMC) and the output signal ranging from 220 to 330 GHz. A horn antenna is connected with the multiplier and illuminates the sample. Meanwhile, an open-ended waveguide probe is utilized to receive the THz waves and connected with a down converter (WR3.4 Mix AMC), which is placed on the moving platform to map the reflected power distributions. The signal analyzer is connected with the down converter to calculate and display the received power. The inset in the upper-right corner shows the zoom-in view of a section of the reflective metasurface prototype.

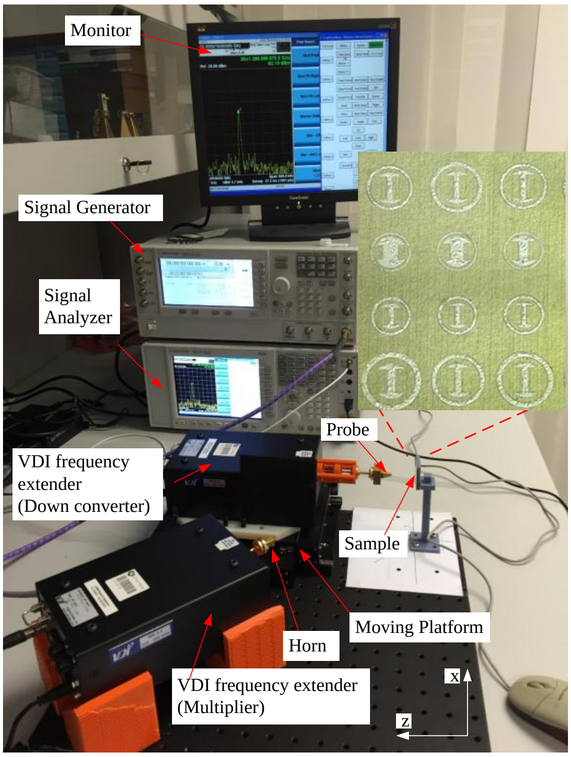

Fig. S3 Photograph of the measurement setup.

IV. Phase responses of elements with different number of resonators

Simple elements with different number of resonators are presented in Figs. S4, S5, and S6. It can be seen that the largest phase differences between 0.2 and 0.3 THz of the elements with one, two, and three resonators are 281°, 532°, and 832°, respectively. These indicate that the phase difference range can be increased by adding more resonators.

One resonator:

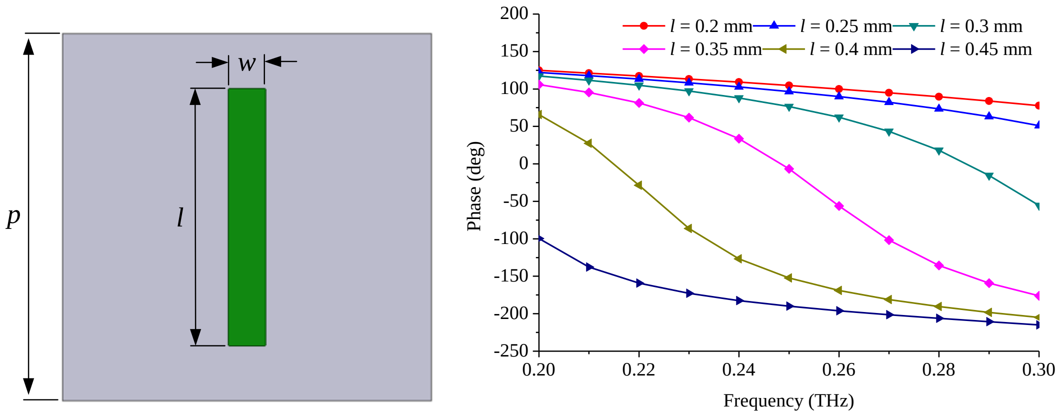

(a) (b)

Fig. S4 (a) Geometry of the element with one resonator. (b) Reflection phase versus frequency with different *l*. *p* = 0.5 mm, *w* = 0.05 mm.

Two resonators:

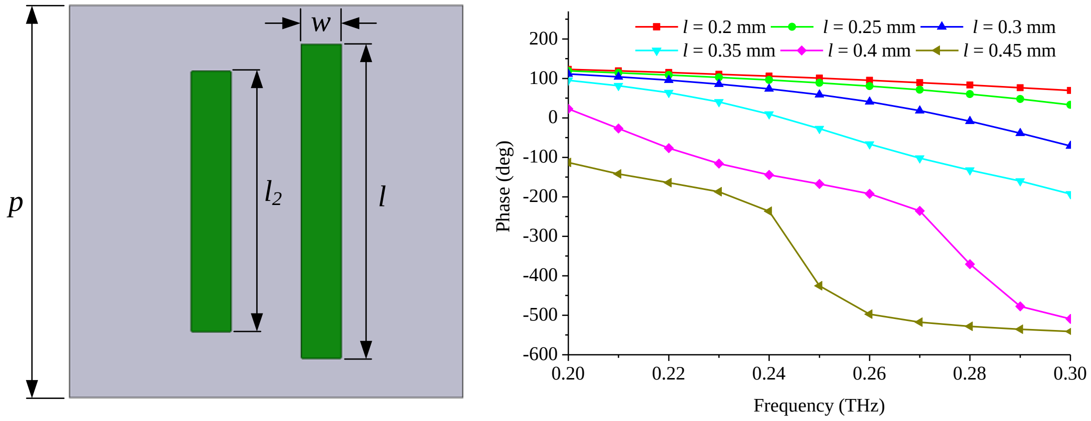

(a) (b)

Fig. S5 (a) Geometry of the element with two resonators. (b) Reflection phase versus frequency with different *l*. *p* = 0.5 mm, *w* = 0.05 mm, $l_2 = 0.83l$.

Three resonators:

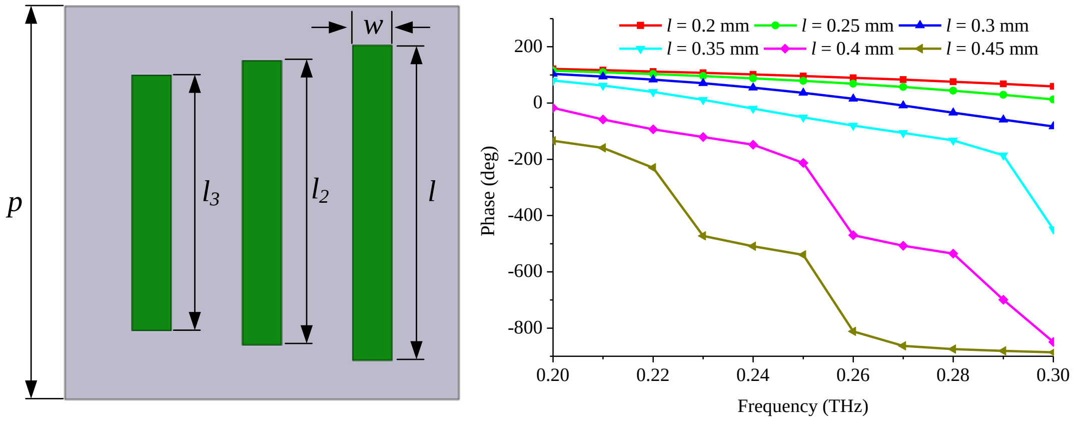

(a) (b)

Fig. S6 (a) Geometry of the element with three resonators. (b) Reflection phase versus frequency with different *l*. *p* = 0.5 mm, *w* = 0.05 mm, $l_2 = 0.9l$, $l_3 = 0.9l_2$.

V. Reflective metasurface with fixed focal position in a certain frequency band

In this design, three frequencies are taken into consideration, i. e., 0.225, 0.250, and 0.275 THz. The corresponding focal points are located at z = 10 mm, with x = y = 0 mm. The desired phase can be calculated using Equation (1) in the main content, and the aforementioned element database is utilized to find the elements that can provide the desired phase compensation at three frequencies simultaneously. The simulated normalized intensity distributions of the reflective metasurface are presented in Fig. S7, it can be seen that the focal length at different frequency is almost fixed. Furthermore, although phase compensation is not taken into consideration at 0.2375 and 0.2625 THz, the focal length is still equal to that at other frequencies.

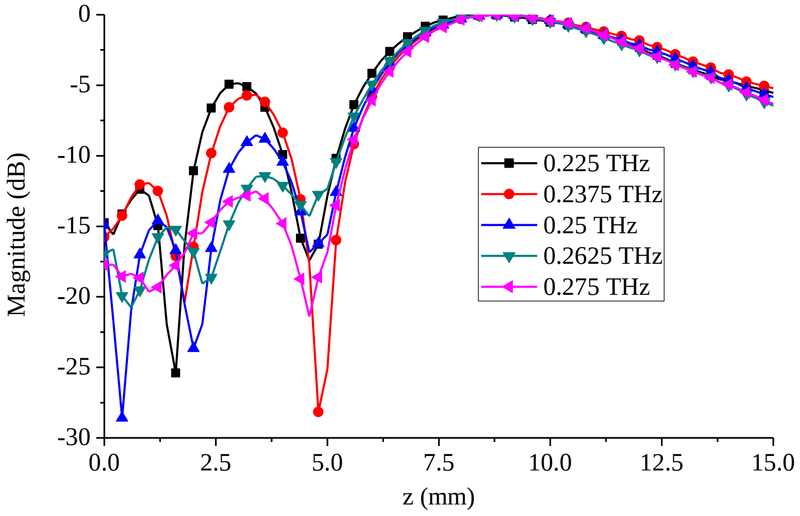

Fig. S7 Simulated normalized intensity distributions of the reflective metasurface with fixed focal length in a certain frequency band. In the figure, the power distribution within a range from 0 to 15 mm is shown. All simulated results are normalized to their maximum values. The simulated results at five frequencies are given, i.e., 0.225, 0.2375, 0.250, 0.2625, and 0.275 THz.

VI. Conversion of the desired phase slope

It can be seen from Figs. 2b and 6a that the desired phase curves exhibit positive slopes in some cases. However, the slopes of the achieved phase curves are negative as presented in Fig. 1c. It seems that the cases which require the phase curves have positive slopes are actually unachievable in physics. In fact, the slopes of the required phase curves can be changed by choosing suitable reference phase $\Phi_0(f)$. For example, if the required phase of the 39th element is regarded as a reference, Fig. 3b can be replotted as presented in Fig. S8. It can be seen that the desired phase curves with positive slopes are adjusted to be negative with the new reference phase.

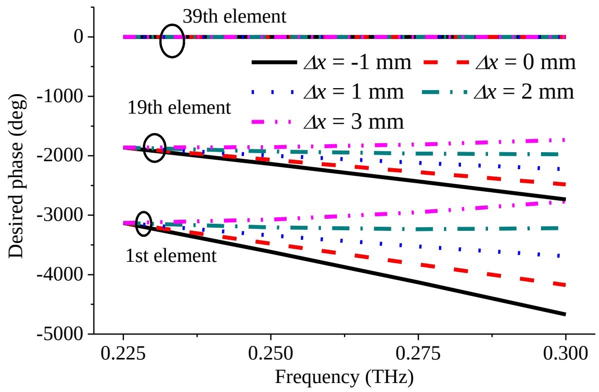

Fig. S8 Desired phases for different positions on the reflective metasurface aperture with different focus-shift spacing. The required phase of the 39th element is regarded as a reference.
